# The First Case of E-Cigarette-Induced Polycythemia

**DOI:** 10.1155/2019/2084325

**Published:** 2019-11-26

**Authors:** Marika Okuni-Watanabe, Keiji Kurata, Kimikazu Yakushijin

**Affiliations:** ^1^Department of Hematology, Kobe City Medical Center West Hospital, 2-4, Ichiban-cho, Nagata-ku, Kobe City, Hyogo 653-0013, Japan; ^2^Department of Medical Oncology and Hematology, Kobe University Hospital, 7-5-2, Kusunoki-cho, Chuo-ku, Kobe City, Hyogo 650-0017, Japan

## Abstract

A 71-year-old male smoker was referred to our hospital because of increased hemoglobin and hematocrit. At initial consultation, his hemoglobin and hematocrit levels were 21.8 g/dl and 64.8%, respectively. Other laboratory data and his cardiopulmonary functions were almost normal, and JAK2 V617F mutation was negative. He had smoked about 25 cigarettes per day for 50 years until the age of 70, when he switched from conventional smoking to electronic cigarettes (e-cigarettes). We requested that he quit e-cigarette use. Thereafter, his hemoglobin and hematocrit gradually decreased and normalized. Here, we report the first case of e-cigarette-induced polycythemia.

## 1. Introduction

Recently, the development of science and technology has been remarkable, with such innovations as the smartphone, tablet computer, electronic money, and virtual reality. These advances successfully enrich our lives; however, they might have unprecedented adverse effects, such as “computer vision syndrome,” which causes users pain in their heads, necks, or shoulders and dry eye syndrome [1].

The electronic cigarette (e-cigarette) is one new invention developed with recent technology; it does not use fire, and is believed to be less hazardous than conventional tobacco. E-cigarettes are battery-operated devices that heat an e-liquid and produce aerosol that the users inhale, while conventional tobacco produces smoke by burning tobacco leaves [2]. E-cigarettes create the simulation of smoking, which can help users to reduce conventional tobacco consumption as they switch from conventional cigarettes to e-cigarettes [3]. It is also known that conventional tobacco may cause many kinds of diseases, one of which is polycythemia [4]; however, there have not yet been any reports on the relationship between e-cigarettes and polycythemia. Here, we report on the first case of e-cigarette-induced polycythemia.

## 2. Case Report

A 71-year-old male smoker was referred to our hospital because of increased hemoglobin and hematocrit. The laboratory data are shown in Table 1. At initial consultation, his hemoglobin and hematocrit levels were 21.8 g/dl and 64.8%, respectively. Eight years earlier, they had been 15.0 g/dl and 43%, respectively. Other laboratory data and his cardiopulmonary functions were approximately normal. Janus kinase 2 (JAK2 V617F) mutation was negative, and erythropoietin was not suppressed. A computed tomography scan revealed no malignancy in his entire body. He had smoked about 25 cigarettes per day for 50 years until he was 70 years old and switched from conventional smoking to e-cigarettes (electronic nonnicotine delivery system, approximately 40 puffs per day) one year before visiting our hospital.

**Figure 1:**
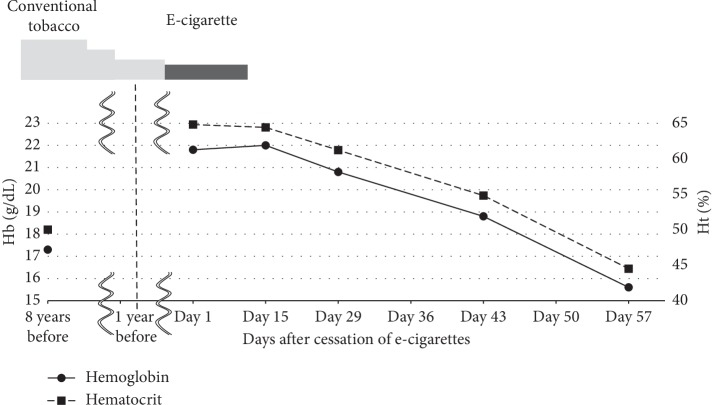
Clinical course. One month after quitting e-cigarette use, his hemoglobin and hematocrit gradually decreased and normalized on day 57.

Speculating about secondary polycythemia, we requested that he quit e-cigarette use. One month later, his hemoglobin and hematocrit had gradually decreased and normalized, as shown in Figure 1. He was diagnosed with e-cigarette-induced polycythemia. Written informed consent for this manuscript was obtained from this patient.

## 3. Discussion

To the best of our knowledge, this is the first report of e-cigarette-induced polycythemia diagnosed by quitting e-cigarette. These days, e-cigarettes have been increasingly popular among smokers who want to stop smoking. As Hajek et al. reported that e-cigarettes could help smokers to quit or reduce tobacco better rather than nicotine replacement therapy [3], their use is likely to increase in the near future. There are hundreds of e-cigarette devices and thousands of e-liquids available globally today, and every e-liquid contains a variety of components and flavors. Therefore, we cannot easily compare various e-cigarettes and do not know much about their safety. However, based on this case, we have doubts about whether e-cigarette use is truly safe.

No research to date has indicated that e-cigarettes cause polycythemia. In this case, polycythemia was thought to be associated with e-cigarette smoking because the patient's hemoglobin and hematocrit levels decreased after quitting e-cigarette smoking. In conventional tobacco cases, carbon monoxide in cigarettes is known to combine with hemoglobin and inhibit hemoglobin from combining with oxygen, which leads to the secretion of erythropoietin and an increase in hemoglobin to compensate for hypoxia. However, it is unclear how e-cigarette use might cause polycythemia, and certain e-cigarettes might involve some carbon monoxide as in a conventional tobacco dose; we speculate that compounds that are only found in e-cigarettes might cause polycythemia because our patient had smoked for decades without polycythemia. It is known that e-cigarettes contain some metals, such as nickel, cadmium, and copper, to some degree [2]. Goldberg et al. suggest that cobalt chloride and nickel chloride seem to stimulate erythropoietin production through the heme protein, which is integrally involved in the oxygen-sensing mechanism using the human hepatoma cell line Hep3B, based on a report that rats with renal-injected nickel showed polycythemia, and nickel could be said to be associated with erythropoietin activity [5]. Thus, Goldberg and colleagues proposed the following hypothesis: cobalt and nickel could be substituted for ferrous iron in the porphyrin ring; however, cobalt hemoglobin binds oxygen with low affinity and nickel hemoglobin is unable to bind oxygen at all. Because of this, the substituted heme protein is locked in the deoxy conformation, mimicking stimulation of deoxygenated heme protein, which stimulates erythropoietin production [5]. Although we did not measure these metals in our patient, we speculate that some of them could be the cause of polycythemia in this case.

Polycythemia is a common disease in the hematology division. While polycythemia vera is known to be the cause of serious thrombosis, it is uncertain whether secondary polycythemia might increase the risk of thrombosis. However, as is it has been reported that secondary polycythemia might cause critical infarction or thrombosis [6–8], it is important to pay attention to e-cigarette smokers' polycythemia.

To date, there have been some reports of e-cigarette-related respiratory disorders, including eosinophilic pneumonitis [9], organizing pneumonia [10], hypersensitive pneumonitis [11], and acute respiratory distress syndrome [12]. Moreover, some researchers doubt the long-term safety of e-cigarettes. Recently, Layden et al. reported 53 cases with pulmonary illness related to e-cigarette use in two states in America [13]. They concluded that e-cigarettes should never be used by youths, young adults, pregnant women, or adults who do not currently use tobacco products although the pathophysiology and definitive causes have not yet been identified [13]. Moreover, Canistro et al. reported that e-cigarettes can raise the risk of cancer [14]. Thus, e-cigarettes might be more harmful than has been believed.

In conclusion, we experienced the first case of “e-cigarette-induced polycythemia,” which was directly clarified by complete hematological improvement after cessation of e-cigarette use. Although new products are generally convenient for human beings, we should reconsider what we are willing to accept from diversified viewpoints.

## Figures and Tables

**Table 1:** Laboratory data.

WBC	5500	/*μ*L
Neutrophils	44	%
Monocytes	7.5	%
Lymphocytes	46	%
Eosinophils	1.5	%
Basophils	0.5	%
Atypical lymphocytes	0.5	%
RBC	705 × 10^4^	/*μ*L
Hb	21.8	g/dL
Ht	64.8	%
Ret	12.9	‰
Plt	11.1 × 10^4^	/*μ*L
		
APTT	27.2	sec
PT-INR	1.06	
D-dimer	<1.0	mg/mL
		
TP	8.06	g/dL
Alb	4.96	g/dL
T-Bil	0.9	mg/dL
AST	39	U/L
ALT	27	U/L
LDH	224	U/L
*γ*-GTP	45	U/L
CK	65	U/L
BUN	12	mg/dL
Cre	0.67	mg/dL
eGFR	88.5	mL/min/1.73m^2^
Na	143	mEq/L
K	4.1	mEq/L
Cl	102	mEq/L
CRP	0.12	mg/dL
Ferritin	233	ng/mL
CEA	3.9	ng/mL
AFP	1.5	ng/mL
CA19-9	5.7	U/mL
Erythropoietin	11.4	mIU/mL
IgG	1382	mg/dL
IgA	227	mg/dL
IgM	162	mg/dL
BNP	24.0	pg/mL
		
JAK2 V617F mutation	Negative	
